# A 1.6 kV Ga_2_O_3_ Schottky Barrier Diode with a Low Reverse Current of 1.2 × 10^−5^ A/cm^2^ Enabled by Field Plates and N Ion-Implantation Edge Termination

**DOI:** 10.3390/nano14110978

**Published:** 2024-06-05

**Authors:** Xinlong Zhou, Jining Yang, Hao Zhang, Yinchi Liu, Genran Xie, Wenjun Liu

**Affiliations:** School of Microelectronics, Fudan University, Shanghai 200433, China; 21212020017@m.fudan.edu.cn (X.Z.); 22112020163@m.fudan.edu.cn (J.Y.); 23112020164@m.fudan.edu.cn (H.Z.); 21112020137@m.fudan.edu.cn (Y.L.); 23212020169@m.fudan.edu.cn (G.X.)

**Keywords:** *β*-Ga_2_O_3_, Schottky barrier diode, field plate, N ion implantation, breakdown voltage

## Abstract

In this work, by employing field plate (FP) and N ion-implantation edge termination (NIET) structure, the electrical performance of the *β*-Ga_2_O_3_ Schottky barrier diode (SBD) was greatly improved. Ten samples of vertical SBDs were fabricated to investigate the influence of the relative positions of field plates (FPs) and ion implantation on the device performance. The device with the FP of 15 μm and the ion implantation at the edge of the Schottky electrode exhibited a breakdown voltage (*V_br_*) of 1616 V, a specific on-resistance (*R_on_*_,*sp*_) of 5.11 mΩ·cm^2^, a power figure of merit (PFOM) of 0.511 GW/cm^2^, and a reverse current density of 1.2 × 10^−5^ A/cm^2^ @ −1000 V. Compared to the control device, although the *R_on_*_,*sp*_ increased by 1 mΩ·cm^2^, the *V_br_* of the device increased by 183% and the PFOM increased by 546.8%. Moreover, the reverse leakage current of the device with the FP and NIET structure decreased by three orders of magnitude. The TCAD simulation revealed that the peak electric field at the interface decreased from 7 MV/cm @ −500 V to 4.18 MV/cm @ −1000 V. These results demonstrate the great potential for the *β*-Ga_2_O_3_ SBD with a FP and NIET structure in power electronic applications.

## 1. Introduction

*β*-Ga_2_O_3_ has garnered significant attention owing to its ultra-wide bandgap of 4.9 eV and high breakdown electric field of 8 MV/cm, making it a promising candidate for next-generation power electronics [1,2]. The development of the large-scale melt-growth technique for the *β*-Ga_2_O_3_ wafer has enabled cost-effective manufacturing approaches [3]. Moreover, thanks to the outstanding chemical and thermal stability of *β*-Ga_2_O_3_ [4,5], Schottky barrier diodes (SBDs) [6,7,8,9] and metal-oxide-semiconductor field-effect transistors (MOSFETs) [10,11,12,13] fabricated on *β*-Ga_2_O_3_ wafers have demonstrated strong competitiveness. However, there remains a significant disparity in the breakdown electric field between the material’s theoretical prediction and the fabricated *β*-Ga_2_O_3_ SBDs. This discrepancy is primarily owing to the material defects and the electric field concentration at the Schottky electrode edge. Various approaches, such as field plates (FPs) [14,15], the trench structure [16,17,18], thermally oxidized termination [19], and heterogeneous termination [20,21], have been proposed to suppress the electric field concentration. Meanwhile, the ion implantation technique [22,23,24,25], which creates a high-resistance region in *β*-Ga_2_O_3_, is considered highly effective for increasing the breakdown voltage (*V_br_*) while reducing the reverse leakage current.

Zhou et al. [22] reported a *β*-Ga_2_O_3_ SBD with Mg-implanted edge termination, achieving a *V_br_* of 1500 V. He and Mg ion implantation were utilized to fabricate *β*-Ga_2_O_3_ SBDs, resulting in a reduced reverse leakage current and enhanced *V_br_* [26]. Yet, the influence of the ion implantation position and the relative position between the FP and ion implantation on the device performance has not been studied, which could have a significant impact on the electrical characteristics.

Here, *β*-Ga_2_O_3_ SBDs with FP and N ion-implantation edge termination (NIET) have been demonstrated. The device with the FP of 15 μm and the ion implantation at the edge of the Schottky electrode improved the *V_br_* from 572 V to 1616 V and achieved the power figure of merit (PFOM) of 0.511 GW/cm^2^. TCAD simulations were employed to analyze the electric field distribution in *β*-Ga_2_O_3_ SBDs, revealing that the FP and NIET structure significantly enhances the device performance.

## 2. Device Structure and Fabrication Process

**Figure 1:**
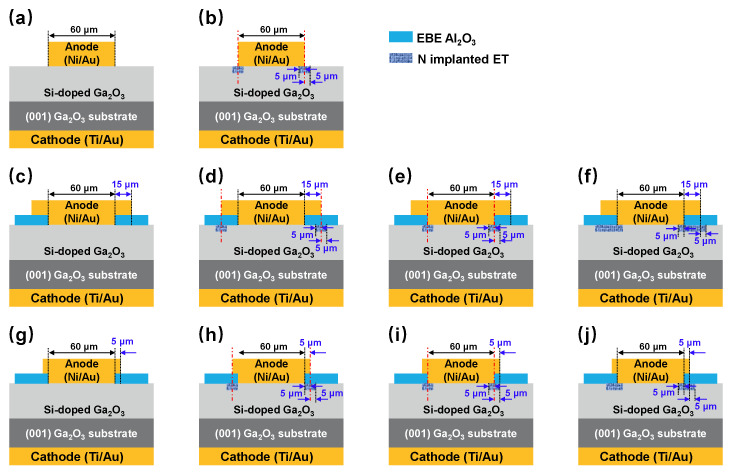
Cross-sectional schematics of (**a**) Sample 1, (**b**) Sample 2, (**c**) Sample 3, (**d**) Sample 4, (**e**) Sample 5, (**f**) Sample 6, (**g**) Sample 7, (**h**) Sample 8, (**i**) Sample 9, and (**j**) Sample 10.

The *β*-Ga_2_O_3_ SBDs were fabricated on a *β*-Ga_2_O_3_ wafer acquired from Novel Crystal Technology (NCT, Tokyo, Japan). The wafer consists of a 587 μm thick *β*-Ga_2_O_3_ (001) substrate with a Sn doping concentration of ~5.1 × 10^18^ cm^−3^ and a 11 μm~3.2 × 10^16^ cm^−3^ Si doping epitaxial layer by halide vapor phase epitaxy (HVPE) [27,28]. To investigate the effect of the FP and NIET structure on the device performance, ten samples, shown in Figure 1, were prepared and categorized into three groups based on the FP dimensions. Group 1, Group 2, and Group 3 had FP lengths of 0, 15, and 5 μm, respectively. Sample 1 served as a control without any special treatment.

**Figure 2:**
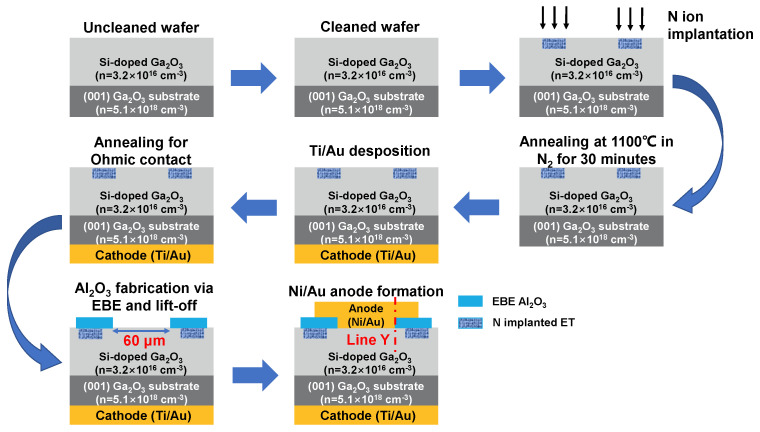
Process flow of the fabricated *β*-Ga_2_O_3_ SBDs with the FP and NIET structure.

**Figure 3:**
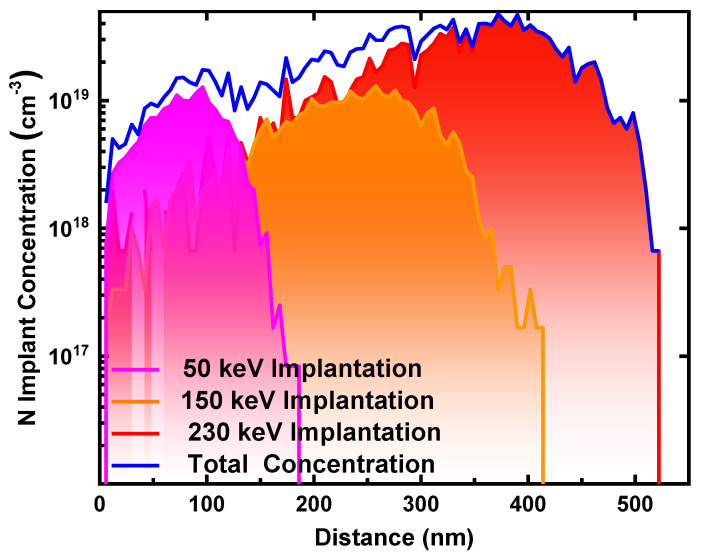
N-implanted total concentration after three N ion implantations.

The device fabrication process is illustrated in Figure 2. Initially, the *β*-Ga_2_O_3_ wafer underwent sequential cleaning with acetone, isopropanol, and deionized (DI) water for 10 min each to eliminate surface contaminants. N ion implantation was performed three times on the wafer to form a 500 nm deep box profile with a simulated doping concentration of around 2~3 × 10^19^ cm^−3^. The implantation was carried out at energies/concentrations of 50 keV/5 × 10^12^ cm^−2^, 150 keV/1 × 10^13^ cm^−2^, and 230 keV/2 × 10^13^ cm^−2^ in sequence, as plotted in Figure 3. Then, thermal annealing was executed at 1100 °C for 30 min in N_2_ to recover the implantation damage and activate the implanted N atoms. The back-Ohmic contact was formed by the electron-beam evaporation (EBE) of Ti/Au (30 nm/100 nm), followed by rapid thermal annealing (RTA) for 60 s at 470 °C in N_2_. Additionally, 100 nm of Al_2_O_3_ was deposited onto the *β*-Ga_2_O_3_ surface via EBE as the FP oxide. The Al_2_O_3_ layer was amorphous. During the process, the vacuum was maintained below 5 × 10^−5^ Pa, and the growth rate was kept at ~0.1 nm/s. Finally, Schottky anode electrodes were fabricated by evaporating Ni/Au (50 nm/100 nm). The forward current–voltage (*I*–*V*) and *V_br_* characteristics were measured using the semiconductor device analyzers Keysight B1500A and B1505A (Santa Rosa, CA, USA), respectively, at room temperature. To evaluate the impact of the NIET on the breakdown performance of the *β*-Ga_2_O_3_ SBD, a physics-based technology computer-aided design (TCAD) simulation in Sentaurus was performed, where the parameters of the *β*-Ga_2_O_3_ in this simulation are shown in Table 1. The Okuto impact ionization model, field-dependent mobility, Shockley Read Hall recombination, and bandgap-narrowing effect were also taken into account.

## 3. Results and Discussion

### 3.1. Electrical Properties and Simulations of Devices in Group 1

**Figure 4:**
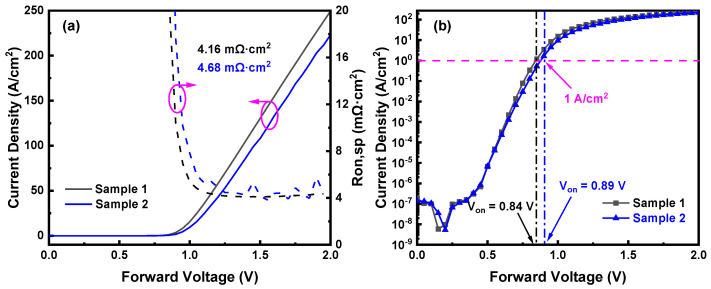
(**a**) Forward *J–V* characteristics and *R_on_*_,*sp*_; (**b**) semi-log scale *J–V* characteristics and *V_on_* of Sample 1 and Sample 2.

Figure 4a shows the linear forward current density–voltage (*J*–*V*) characteristics of Sample 1 and Sample 2. The specific on-resistances (*R_on_*_,*sp*_) of Sample 1 and Sample 2 are 4.16 mΩ·cm^2^ and 4.68 mΩ·cm^2^, respectively. The increase in the *R_on_*_,*sp*_ post-ion implantation is attributed to the N-implanted Ga_2_O_3_ acting as a current-blocking layer [29,30], thereby reducing the effective area of the Schottky contact. Figure 4b depicts the semi-logarithmic *J*–*V* curves of Sample 1 and Sample 2, where the turn-on voltage (*V_on_*) is extracted at the current density of 1 A/cm^2^. The *V_on_* also increases from 0.84 V to 0.89 V by ion implantation. This could be mainly due to the lateral depletion problem of edge termination [26].

**Figure 5:**
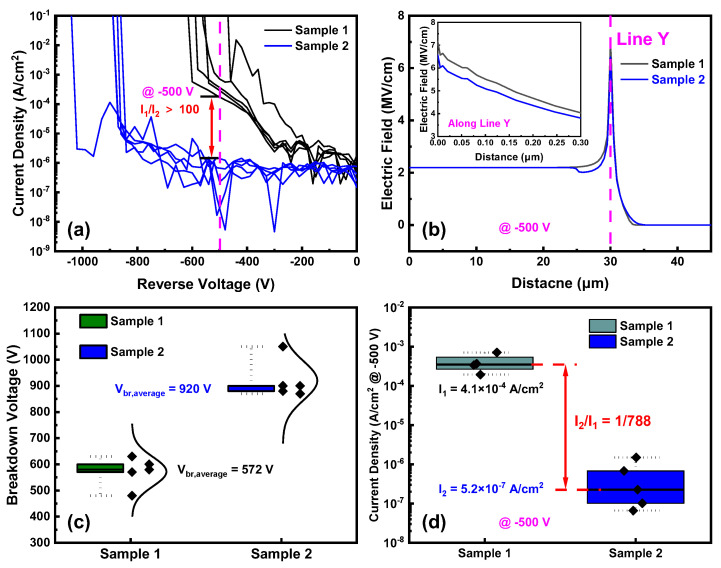
(**a**) Reverse characteristics; (**b**) simulations of the surface electric field distributions; (**c**) statistical plots of *V_br_*; (**d**) statistical plots of the reverse leakage current densities of Sample 1 and Sample 2.

Figure 5a presents the reverse breakdown characteristics, indicating that Sample 2 has a higher *V_br_* and significantly lower leakage current compared to Sample 1. The leakage current of Sample 2 is less than 1/100th of that of Sample 1 at −500 V. Figure 5b shows the surface electric field distributions of Sample 1 and Sample 2 simulated by the TCAD. The implanted region with a depth of 500 nm is set as the semi-insulating layer because the crystal lattice is damaged by the high-energy N ion implant. The peak electric fields of both samples are located at 30 μm, the edge of the Schottky electrode. Additionally, the vertical electric field distributions along Line Y, which is shown in Figure 2, are plotted in the inset of Figure 5b. The right half of the device is set as the simulation cell, with the center of the device as the origin. The positive x-axis extends to the right, parallel to the material surface, and the positive y-axis extends downward, perpendicular to the material surface. The peak electric field of Sample 1 exceeds 7 MV/cm at −500 V, while, for Sample 2, it is around 6.5 MV/cm. These results show that the ion-implantation ET structure can reduce the peak electric field at the interface and prevent premature device breakdown. Figure 5c,d display the statistical distributions of the reverse breakdown and the leakage current @ −500 V for Sample 1 and Sample 2. The average breakdown voltages of Sample 1 and Sample 2 are 572 V and 920 V, respectively, illustrating a 60.8% increase after ion implantation, which is mainly owing to the electric field concentration suppressed with the ion-implanted ET structure. On the other hand, the average leakage current density @ −500 V of Sample 2 is 5.2 × 10^−7^ A/cm^2^, an approximately nearly three orders of magnitude reduction in the reverse leakage current.

### 3.2. Electrical Properties and Simulations of Devices in Group 2

**Figure 6:**
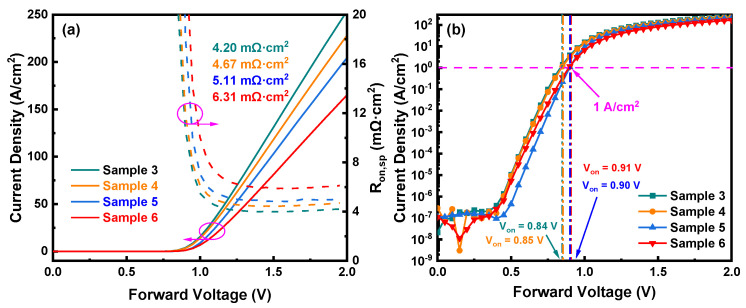
(**a**) Forward *J–V* characteristics and *R_on_*_,*sp*_; (**b**) semi-log scale *J*–*V* characteristics and *V_on_* of Sample 3 to Sample 6.

Figure 6a depicts the forward *J*–*V* characteristics of Sample 3 to Sample 6, where the specific on-resistances of Sample 3, Sample 4, Sample 5, and Sample 6 are 4.20 mΩ·cm^2^, 4.67 mΩ·cm^2^, 5.11 mΩ·cm^2^, and 6.31 mΩ·cm^2^, respectively. Sample 6 shows a 23.4% decrease in the *R_on_*_,*sp*_ compared to Sample 5, which could be ascribed to the fact that a larger ion-implanted region of Sample 6 leads to more N ion diffusion after annealing. The semi-logarithmic *J–V* characteristics of Sample 3 to Sample 6 are shown in Figure 6b. The turn-on vlotages of Sample 3, Sample 4, Sample 5, and Sample 6 are 0.84 V, 0.85 V, 0.90 V, and 0.91 V, respectively, where the turn-on voltages of Sample 5 and Sample 6 show a more significant increase. This implies that the ion implantation close to the edge of the Schottky electrode has a greater impact on the *V_on_* of the devices.

**Figure 7:**
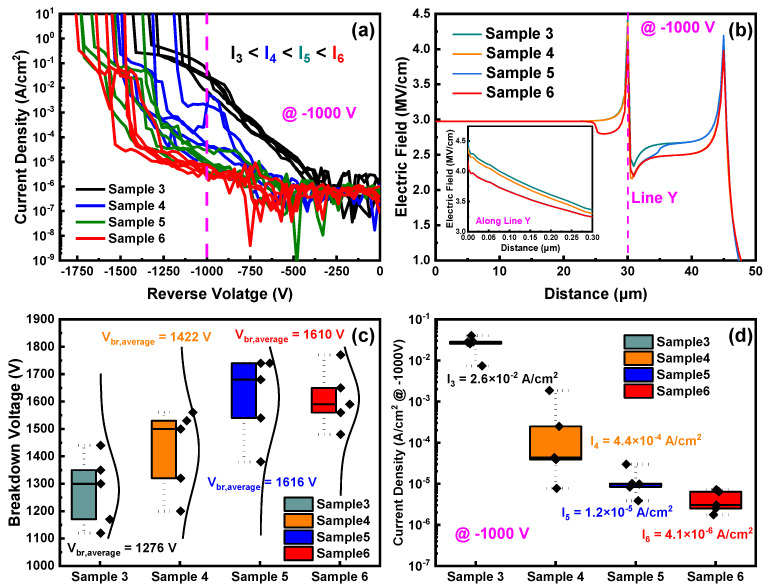
(**a**) Reverse characteristics; (**b**) simulations of the surface electric field distributions; (**c**) statistical plots of *V_br_*; (**d**) statistical plots of reverse leakage current densities of Sample 3 to Sample 6.

Figure 7a conveys the reverse breakdown characteristics of Sample 3 to Sample 6. Under the same reverse bias, Sample 3 has a significantly higher leakage current than other samples, confirming that the combined FP and NIET structure results in lower reverse leakage current compared to the conventional FP structure. Figure 7b shows the surface electric field distributions of Sample 3 to Sample 6, with two peak positions at 30 μm and 45 μm, corresponding to the edge of the Schottky electrode and the edge of the FP, respectively. The higher electric field at 30 μm can trigger premature breakdown. The inset displays the vertical electric field along Line Y, revealing that, at 30 μm, the peak electric fields of Sample 5 and Sample 6 are nearly equivalent and significantly lower than those of Sample 3 and Sample 4. This demonstrates that ion implantation at the edge of the Schottky electrode in Sample 5 and Sample 6 effectively suppresses the peak electric field. Meanwhile, the peak electric field of Sample 4 is slightly lower than that of Sample 3, indicating that ion implantation at the edge of the FP has little effect on the peak electric field. Figure 7c,d plot the statistical distributions of *V_br_* and the reverse leakage current density @ −1000 V for Sample 3 to Sample 6. The average breakdown voltages for Sample 3, Sample 4, Sample 5, and Sample 6 are 1276 V, 1422 V, 1616 V, and 1610 V, respectively, while the average leakage current densities are 2.6 × 10^−2^ A/cm^2^, 4.4 × 10^−4^ A/cm^2^, 1.2 × 10^−5^ A/cm^2^, and 4.1 × 10^−6^ A/cm^2^, respectively. These results suggest that the FP and NIET structure significantly improves the reverse characteristics, with ion implantation at the Schottky electrode edge offering greater enhancement compared to implantation at the FP edge.

### 3.3. Electrical Properties and Simulations of Devices in Group 3

**Figure 8:**
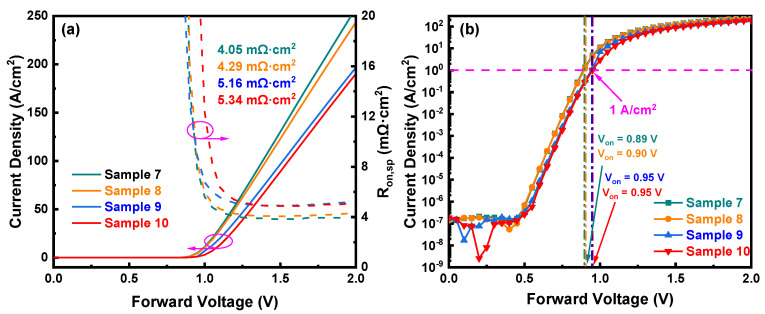
(**a**) Forward *J*–*V* characteristics and *R_on_*_,*sp*_; (**b**) semi-log scale *J*–*V* characteristics and *V_on_* of Sample 7 to Sample 10.

Figure 8a depicts the linear forward *J*–*V* characteristics of Sample 7 to Sample 10, with specific on-resistances of 4.05 mΩ·cm^2^, 4.29 mΩ·cm^2^, 5.16 mΩ·cm^2^, and 5.34 mΩ·cm^2^, respectively. The *R_on_*_,*sp*_ of Sample 4 increased by 0.47 mΩ·cm² compared to Sample 3, while the *R_on_*_,*sp*_ of Sample 8 increased by 0.24 mΩ·cm² compared to Sample 7. The smaller increase in *R_on_*_,*sp*_ for Sample 8 compared to Sample 7 could be mainly caused by the current crowding in the 5 μm FP and NIET composite structure, which results in a lower *R_on_*_,*sp*_ for the device. Figure 8b presents the semi-logarithmic *J*–*V* curves of Sample 7 to Sample 10, with turn-on voltages of 0.89 V, 0.90 V, 0.95 V, and 0.95 V, respectively. The *R_on_*_,*sp*_ and *V_on_* of the samples in Group 3 exhibit analogous tendencies to those in Group 2, indicative of a positive correlation between *V_on_* and the distance from the ion implantation position to the Schottky contact area, as well as the ion implantation area.

**Figure 9:**
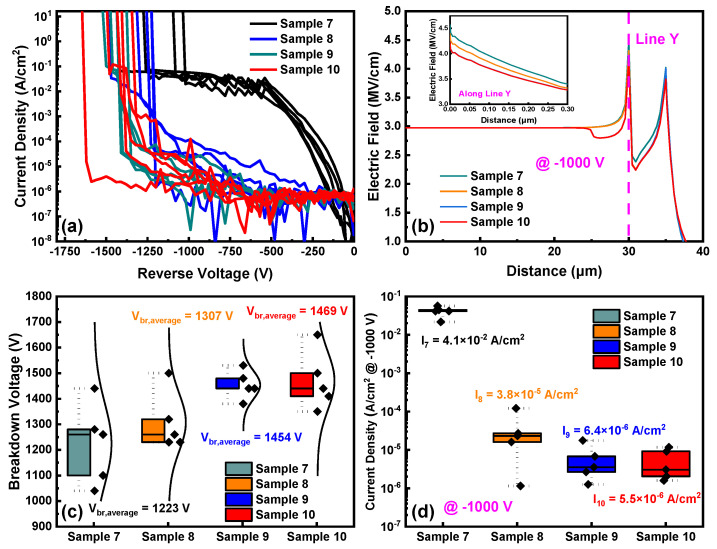
(**a**) Reverse characteristics; (**b**) simulations of the surface electric field distributions; (**c**) statistical plots of *V_br_*; (**d**) statistical plots of reverse leakage current densities of Sample 7 to Sample 10.

Figure 9a presents the reverse breakdown characteristics of Sample 7 to Sample 10, showing that Sample 7 has a significantly higher leakage current than the other samples, consistent with the results of the Group 2. Figure 9b displays the surface electric field distributions of Sample 7 to Sample 10, with the inset portraying the vertical electric field along Line Y. The electric field at 30 μm remains higher than that at 45 μm. Figure 9c,d depict the *V_br_* and the reverse leakage current density @ −1000 V statistics for Sample 7 to Sample 10, with average breakdown voltages of 1223 V, 1307 V, 1454 V, and 1469 V, respectively, and average leakage current densities of 4.1 × 10^−2^ A/cm^2^, 3.8 × 10^−5^ A/cm^2^, 6.4 × 10^−6^ A/cm^2^, and 5.5 × 10^−6^ A/cm^2^, respectively. The tendencies of the *V_br_* and reverse leakage current densities of the samples in Group 3 are consistent with those in Group 2. Additionally, the breakdown voltages of Sample 5 and Sample 6 are higher than those of Sample 9 and Sample 10. This reveals that ion implantation at the edge of the Schottky electrode enhances *V_br_* more effectively when the FP length is 15 μm. 

### 3.4. Comprehensive Comparisons and Analysis

**Figure 10:**
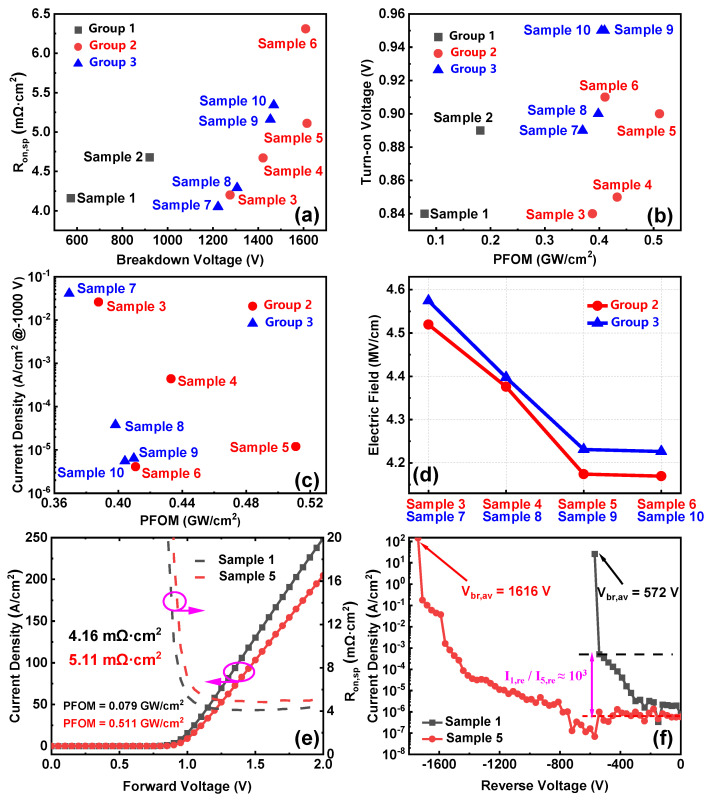
Benchmark plots of (**a**) *R_on_*_,*sp*_ vs. *V_br_*, (**b**) *V_on_* vs. PFOM, (**c**) and reverse leakage current densities @ −1000 V vs. PFOM. (**d**) The peak electric fields of Group 2 and Group 3. (**e**) Forward *J–V* characteristics. (**f**) Reverse characteristics of Sample 1 and Sample 5.

Table 2 summarizes the *V_br_*, *R_on_*_,*sp*_, *V_on_*, and reverse leakage current densities (*J_rev_*) of Samples 1–10. Figure 10a–c show the benchmark plots of *R_on_*_,*sp*_ versus *V_br_*, *V_on_* versus PFOM, and *J_rev_* versus PFOM, respectively. Sample 5 achieves the highest PFOM, with a very low reverse leakage current of 1.2 × 10^−5^ A/cm^2^ @ −1000 V, although it has a relatively high *V_on_*. On the other hand, Sample 4 shows little increase in *V_on_* compared to the conventional device; however, its reverse characteristics are inferior to Sample 5. Figure 10d sums up the simulated peak electric fields of Group 2 and Group 3, where the former is generally lower than the latter, suggesting that the devices in Group 2 exhibit superior breakdown tolerance compared to the devices in Group 3. Ion implantation with an FP length of 15 μm significantly improves device electrical performance. Figure 10e,f compare the electrical characteristics of Sample 1 and Sample 5, showing that the *β*-Ga_2_O_3_ SBD with the FP and NIET structure shows a 183% increase in *V_br_* and a reduction in the reverse leakage current by three orders of magnitude relative to the conventional device. Although the *R_on_*_,*sp*_ increases by 1 mΩ·cm^2^, the PFOM of the device increases by 546.8%.

## 4. Conclusions

In summary, we have examined the effects of the FP and NIET structure on the performance of *β*-Ga_2_O_3_ SBDs. The TCAD simulation suggests that the surface peak electric field of the sample is significantly decreased by the FP and NIET structure. The device with the FP and NIET structure achieves a *V_br_* of 1616 V, a *R_on_*_,*sp*_ of 5.11 mΩ·cm^2^, a PFOM of 0.511 GW/cm^2^, and a reverse leakage current density of 1.2 × 10^−5^ A/cm^2^ @ −1000 V. These results demonstrate that the FP and NIET structure is highly promising for boosting the performance of *β*-Ga_2_O_3_ power electronics.

## Figures and Tables

**Table 1:** Material parameters used in this simulation.

Parameters	*β*-Ga_2_O_3_
Bandgap (eV)	4.8
Affinity (eV)	4.0
*N_C_* (cm^−3^)	3.7 × 10^18^
*N_V_* (cm^−3^)	5.0 × 10^18^
Relative permittivity	10.0
Electron mobility (cm^2^ V^−1^ s^−1^)	200
Saturation velocity (cm s^−1^)	2 × 10^7^

**Table 2:** *V_br_*, *R_on_*_,*sp*_, *V_on_*, and reverse leakage current densities of Samples 1–10.

Device	*V_br_* (V)	*R_on_*_,*sp*_ (mΩ·cm^2^)	*V_on_* (V)	*J_rev_* (A/cm^2^ @ −1000 V)
Sample 1	572	4.16	0.84	
Sample 2	920	4.68	0.89	
Sample 3	1276	4.20	0.84	2.6 × 10^−2^
Sample 4	1422	4.67	0.85	4.4 × 10^−4^
Sample 5	1616	5.11	0.90	1.2 × 10^−5^
Sample 6	1610	6.31	0.91	4.1 × 10^−6^
Sample 7	1223	4.05	0.89	4.1 × 10^−2^
Sample 8	1307	4.29	0.90	3.8 × 10^−5^
Sample 9	1454	5.16	0.95	6.4 × 10^−6^
Sample 10	1469	5.34	0.95	5.5 × 10^−6^

## Data Availability

Data are contained within the article.
